# Importance of the endometrial immune environment in endometrial cancer and associated therapies

**DOI:** 10.3389/fonc.2022.975201

**Published:** 2022-08-22

**Authors:** Hannah van der Woude, Kathryn Elizabeth Hally, Margaret Jane Currie, Olivier Gasser, Claire Elizabeth Henry

**Affiliations:** ^1^ Department of Obstetrics, Gynaecology and Women’s Health, University of Otago, Wellington, New Zealand; ^2^ Department of Surgery and Anesthesia, University of Otago, Wellington, New Zealand; ^3^ Department of Pathology and Biomedical Science, University of Otago, Christchurch, New Zealand; ^4^ Malaghan Institute of Medical Research, Wellington, New Zealand

**Keywords:** endometrial cancer, immunotherapy, microenvironment, adiposity, levonorgestrel

## Abstract

Endometrial cancer is rising in prevalence. The standard treatment modality of hysterectomy is becoming increasingly inadequate due primarily to the direct link between endometrial cancer and high BMI which increases surgical risks. This is an immunogenic cancer, with unique molecular subtypes associated with differential immune infiltration. Despite the immunogenicity of endometrial cancer, there is limited pre-clinical and clinical evidence of the function of immune cells in both the normal and cancerous endometrium. Immune checkpoint inhibitors for endometrial cancer are the most well studied type of immune therapy but these are not currently used as standard-of-care and importantly, they represent only one method of immune manipulation. There is limited evidence regarding the use of other immunotherapies as surgical adjuvants or alternatives. Levonorgestrel-loaded intra-uterine systems can also be effective for early-stage disease, but with varying success. There is currently no known reason as to what predisposes some patients to respond while others do not. As hormones can directly influence immune cell function, it is worth investigating the immune compartment in this context. This review assesses the immunological components of the endometrium and describes how the immune microenvironment changes with hormones, obesity, and in progression to malignancy. It also describes the importance of investigating novel pathways for immunotherapy.

## Introduction

Endometrial cancer (EC), or cancer originating in the uterine epithelium, is the most prevalent gynecological cancer in the developed world (1, 2). Rates of EC are rising, and prevalence is increasing in younger people. This is due, in part, to the rise in obesity; increased adiposity raises estrogen production and adipokine release which results in an oncogenic signal, stimulating endometrial cell proliferation (3). The predominant treatment for EC is hysterectomy which sometimes includes bilateral salpingo oophorectomy and pelvic lymph node dissection. Late-stage disease can also receive adjuvant chemotherapy or radiation. While this treatment pathway is effective, hysterectomy is an invasive procedure for early-stage disease which removes the fertility of the patient and is high-risk for those with a high body mass index (BMI). The use of the levonorgestrel-loaded intra-uterine system (LNG-IUS) circumvents these issues in patients who respond, however, response rates to LNG-IUS treatment for EC are as low as 40% (4). In EC, as with many cancers, the tumor microenvironment plays a significant role in cancer progression and response to therapy. This includes both the interaction between the tumor and stroma and the interaction between the tumor and infiltrating immune cells. Immune cells in the normal endometrium play important roles in protection from external pathogens, aiding fertilization, and tolerance and maintenance of pregnancy. Significant infiltration of immune cells characterize certain subtypes of EC, suggesting that immunotherapies may be effective as therapeutic alternatives or adjuvants to surgery in a subgroup of patients. As such, the literature examining the immunological tumor microenvironment (iTME) of EC has focused on the potential for specific EC subtypes to respond to immunotherapy. Within this literature, emphasis has been placed on examining immune checkpoint inhibitors (ICI). However, ICI success is variable and most ICIs for treatment of EC are still in clinical trial stage. A summary of this literature has been recently reviewed by Cao et al. (5). Investigating alternative methods of immunomodulation could increase the scope of therapies available, making personalized treatment for EC more feasible. A more holistic understanding of the composition of EC-infiltrating immune cells and their function within the iTME is warranted and could provide evidential support for the use of other types of immunotherapies in this context. Here, we aim to review the evidence describing the iTME of EC and how this may be influenced by increasing adiposity, discuss the successes and pitfalls of immunotherapies for EC treatment, and provide recommendations to fill the knowledge gaps that exist within this body of literature.

## Endometrial cancer classification

The pathogenesis of EC is the over-proliferation of endometrial glands resulting in an abnormal gland-to-stroma ratio (6). Currently, diagnosis and classification of EC is based largely on the histological phenotype of the tumor cells biopsied by pipelle or curettage and, following surgical intervention, on the primary tumor. Histological subtypes include endometrioid, clear cell, serous, and mucinous. Endometrioid endometrial carcinoma (EEC) is the most common subtype, accounting for between 75 – 90% of cases (7, 8). EEC is strongly associated with prolonged estrogen exposure and has the best prognosis due to its often early presentation with abnormal bleeding (9). Serous carcinomas contribute to approximately 10% of EC cases (10), whereas clear cell and mucinous endometrial carcinomas are rare, collectively contributing to less than 5% (9).

In addition to histological grading, The Cancer Genome Atlas (TCGA) categorized EC into four prognostically distinct molecular subtypes using whole genome sequencing, irrespective of histology (11). These are: microsatellite instability high (MSI-H), DNA polymerase ϵ (*POLE*) mutated, copy number low, and copy number high (11). *POLE* mutated tumors have the highest progression-free survival rates, while copy number low and MSI-H tumors are intermediate risk and copy number high tumors have the poorest prognosis (11). Diagnostic testing must be resource, time and cost-effective, and whole genome sequencing does not fall into these parameters. To combat this, Talhouk et al. extrapolated TCGA classifications into groups that are defined using a combination of immunohistochemistry (IHC) markers and targeted DNA sequencing, creating a ‘proactive molecular risk classifier for endometrial cancer’, or ProMisE (12). The four molecular subtypes thus became: mismatch repair deficient (MMRd), *POLE* exonuclease domain mutant (*POLE*mut), p53 wild type/nonspecific molecular profile (NSMP), and p53 abnormal (p53abn). Since their conception, validation and confirmation of the ProMisE molecular subtypes has been conducted to identify whether these subtypes have different therapeutic outcomes. This topic has been recently comprehensively reviewed by Mitric and Bernardini (13). For example, the PORTEC3 clinical trial investigated the benefit of adjuvant combined chemotherapy and radiotherapy compared to chemotherapy alone in patients with high grade and/or stage endometrial cancer (14). Subsequent analysis separated trial participants into molecular subtype based on the ProMisE guidelines (12) and found a significant benefit for patients with p53abn tumors receiving combined chemoradiotherapy compared to chemotherapy alone (*P* = 0.019) (15). Furthermore, those with *POLE*mut EC had an excellent recurrence free survival regardless of treatment (15).

## Immunity in the normal endometrium

To better understand the immunological characteristics of EC, it is important to contextualize the role of immune cells within the homeostatic immune interactions of the normal endometrium. The healthy endometrial immune environment is characterized by cyclic shifts in immune cell proportions and functionality due to hormonal changes throughout the menstrual cycle and its direct exposure to environmental pathogens and the uterine microbiota. Local immune cells protect against pathogens and support endometrial remodeling during menstruation, conception, and pregnancy (16). Reproductive hormones are likely to be the dominant factor driving immunoregulation in the endometrium, as these hormones generally induce immunological suppression to facilitate conception. This idea has been reviewed extensively elsewhere (17). In non-immune cells, the estrogen receptor (ER) and progesterone receptor (PR) are intracellularly located and function as transcription factors. While expression of ER by immune subsets is widely accepted (18, 19), the exact mechanism of progesterone immunomodulation is not fully understood. Early studies of endometrial-derived immune cells show no overlap of CD45 and PR expression (20–22), and RNA sequencing has found no detectable nuclear PR expression in T cells (23). Notwithstanding, progesterone demonstrably influences T cell function, observed by a decrease in T cell expression of the inflammatory cytokines interferon-γ (IFN-γ), and tumor necrosis factor-α (TNF-α), and an increase in expression of the regulatory cytokine IL-4 (23). Progesterone also hinders T cell proliferation and activation (24). T cell-intrinsic expression of PR therefore remains the prevailing paradigm (25–28). Alternative mechanisms of progesterone signaling include membrane progesterone receptors, indirect signaling *via* stromal cells, and signaling *via* the glucocorticoid receptor (23, 29–31).

**Figure 1:**
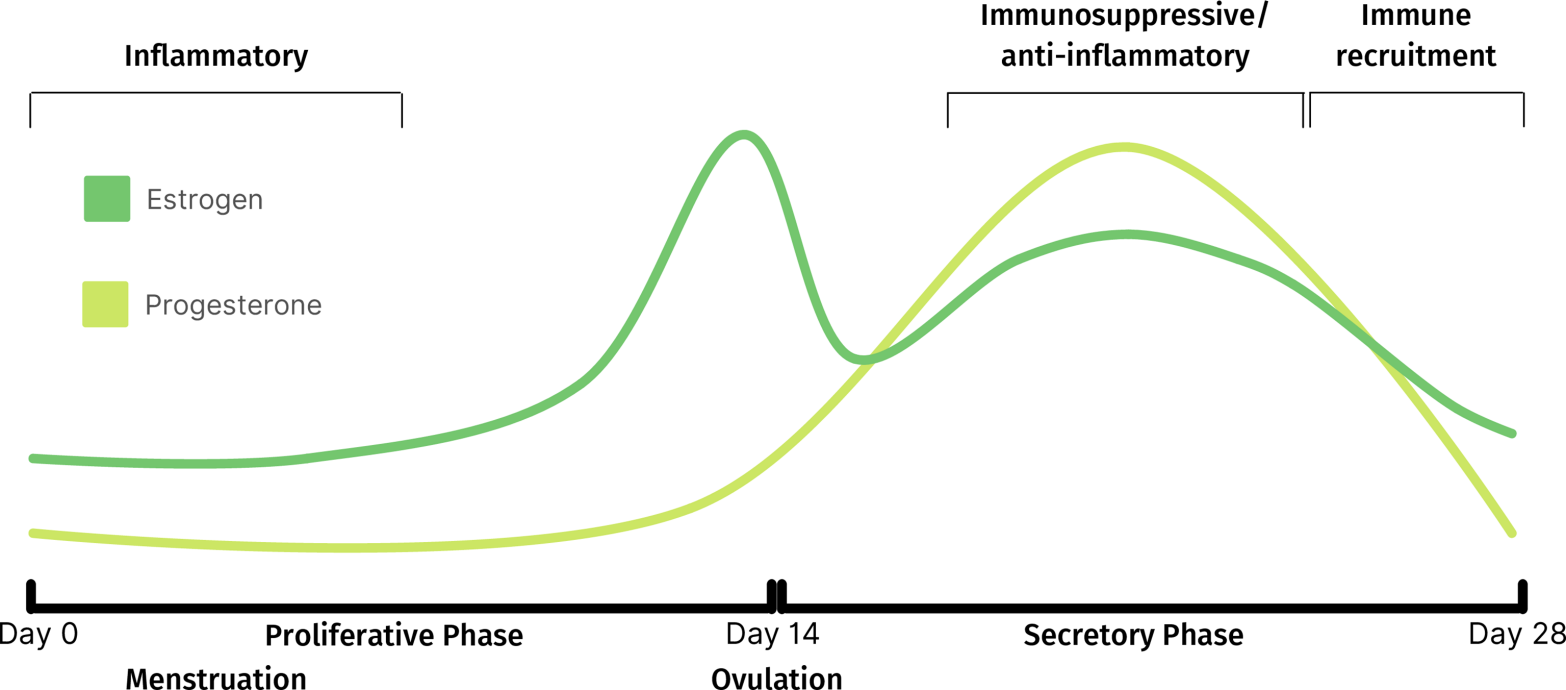
Fluctuations of estrogen and progesterone throughout normal menstrual cycle and corresponding immune activity.

Progesterone, which begins to increase following ovulation (**Figure 1**), exerts immunosuppressive activity through intrinsic and extrinsic mechanisms to regulate immune cell function and trafficking, respectively (24, 32). For example, the release of the cytolytic molecule, perforin, from CD56^+^ cells is inhibited by progesterone (32). Additionally, the declining levels of progesterone towards the late-secretory phase triggers a pro-inflammatory signaling cascade which leads to macrophage and neutrophil recruitment and the release of degradative enzymes required for menstruation. The resulting sterile inflammation and tissue remodeling processes, triggered during the decline of progesterone, are tightly regulated to promote scarless healing (33, 34). The environment then switches from pro- to anti-inflammatory as the cycle continues (35–37). There is also evidence suggesting that the uterine microbiota may play a role in immune regulation (38).

**Figure 2:**
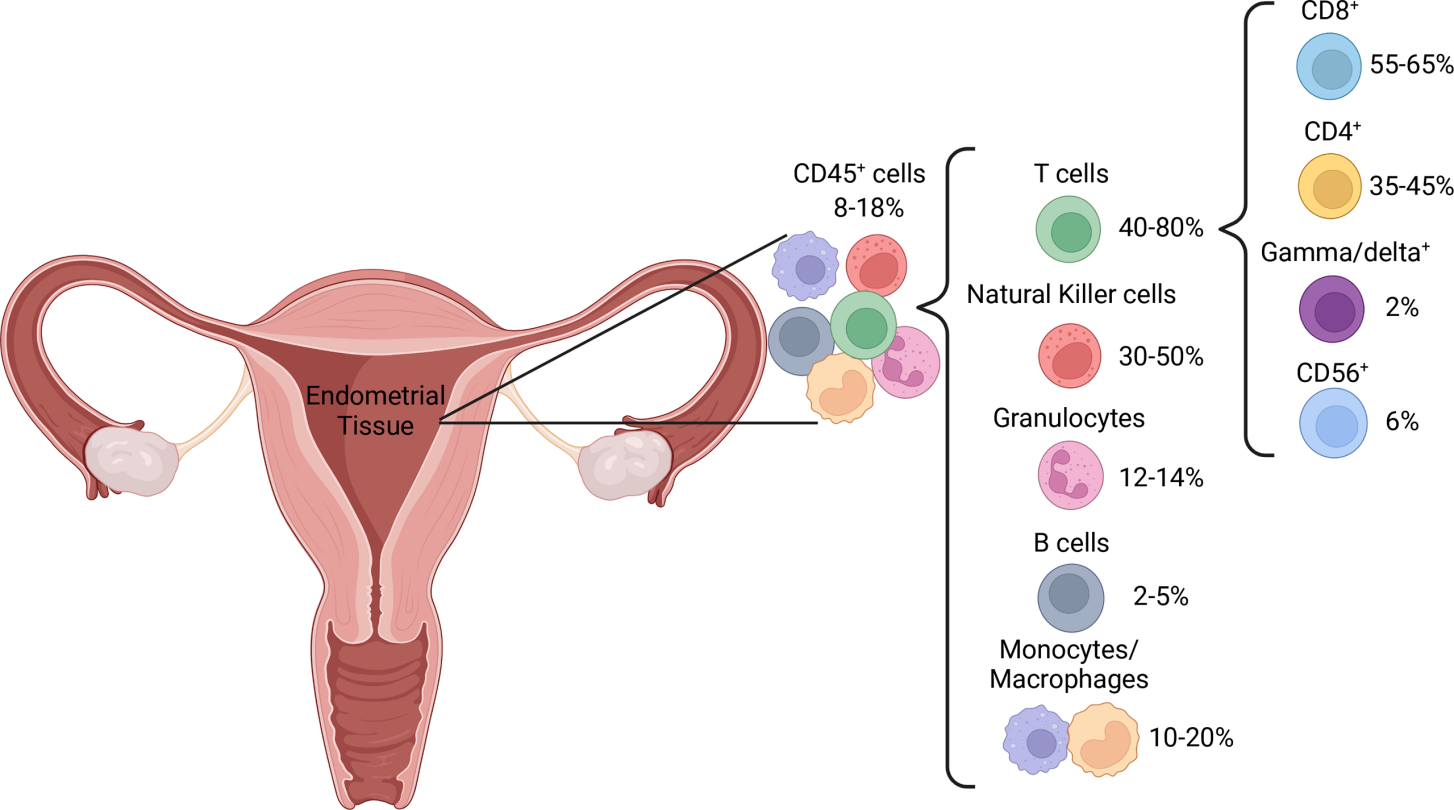
Distribution of immune cell populations in normal endometrial tissue.

Pre-menopause, the proportion of immune cells within the endometrium fluctuates with the menstrual cycle, which has led to variable reports of immune cell distribution. The proportion of T cells is higher in the proliferative, compared to the secretory, phase (39). This is likely due to a large increase in NK cell numbers (40) rather than a decrease in T cell numbers, as absolute T cell counts are stable throughout the menstrual cycle (16, 41). Post-menopausal uteri have similar T cell proportions to those in the proliferative phase but contain a higher proportion of granulocytes (39), potentially as a result of endometrial atrophy characteristic of menopausal uteri. T cells are the dominant immune cell subset throughout the female reproductive tract, including the endometrium, where they represent 1-5% of all endometrial tissue cells (39, 42) and 40-80% of CD45^+^ immune cells, depending on menstrual cycle stage (39, 43) (**Figure 2**). In addition to residing within the epithelium and being scattered amongst stromal cells, endometrial immune cells form lymphoid aggregates (LAs). LAs are comprised of a B cell core surrounded by CD8^+^ T cells, which are themselves surrounded by macrophages and some NK cells (44). LAs begin to develop during the end of the proliferative phase, becoming larger during the secretory phase, and are absent post-menopause (45). While the existence of LAs is well established in early research (44–47), recent research confirming their existence is lacking, and their function remains unclear. The majority of endometrial T cells express the co-receptor CD8, which is likely due to the prominence of CD8^+^ T cells within LAs (44, 48, 49). However, the effector functions of endometrial CD8^+^ T cells may extend beyond their traditional function of cytotoxicity, as reflected by the functional plasticity displayed by decidual CD8^+^ T cells during pregnancy (50). Moreover, CD3^+^ T cells obtained from the endometrium during the secretory phase exhibit limited cytotoxic capacity compared to those obtained during the proliferative phase (51). This suggests that the LAs, which develop during the secretory phase, play a regulatory role that coincides with potential blastocyst implantation. It has also been postulated that the presence of LAs in the basalis stroma, an inner portion of the endometrium that is not shed during menstruation, is a means of maintaining immune presence during menstruation (52). This would allow the immune system to quickly re-infiltrate the regenerating endometrium post-menstruation.

Endometrial T cells also include a significant proportion of CD4^+^ T cells, at approximately 40% of T cells (40). CD4^+^ T cells, also known as helper T cells, play an integral role in the immune response by activating other effector cells such as macrophages, CD8^+^ T cells and B cells (53). The function of endometrial CD4^+^ T cells is less well established than their CD8^+^ counterparts, however the balance of the CD4^+^ T cell subsets Th1/Th2/Th17/Treg appears to be important in maintaining pregnancy (54), and this balance must be present prior to conception (55). For example, the pre-pregnant and pregnant endometrium is more inclined towards a Th1 phenotype than a Th2 phenotype (55), and a decrease in the proportion of Th1 CD4^+^ T cells in the endometrium could lead to recurrent miscarriage (56). Also present in the endometrium are γδ^+^ and CD3^+^CD56^+^ T cells (40), referred to as innate-like T cells due to their non-specific nature and functional similarity to components of the innate immune system (57). These cells may provide additional regulatory tone. Additionally, decidual CD3^-^CD56^+^ NK cells express high levels of CD56 (58). There is a significant increase in CD56^bright^ cells during the periovulatory period, which is thought to occur through NK cell trafficking from peripheral lymphoid tissues, as well as proliferation in the uterine mucosa (59, 60). As CD56^bright^ NK cells are believed to represent the immature and regulatory precursors of their cytotoxic CD56^dim^ counterparts (61), it is likely that the endometrial NK cell compartment favors cytokine production and regulatory functions over cytotoxicity. Increasing levels of estrogen and luteinizing hormone prior to ovulation increases adhesion of natural killer (NK) cells in the uterus (59). This is also thought to be a necessary precursor to successful pregnancy, as these decidual NK cells play important roles in tissue remodeling, embryonic development, trophoblast invasion and placentation, and are seldom cytotoxic unless primed by pathogens (62).

The phagocytic cells of the innate immune system – granulocytes, monocytes and macrophages, collectively make up 25% of the CD45^+^ endometrial immune milieu, whereas B cells are comparably rare regardless of hormonal cycle stage, at no more than 5% (17, 39, 43, 63). Endometrial B cells exist primarily within LAs, but have also been detected in the stroma (45, 64). The function of B cells within the endometrium is not well established, as was concluded in a systematic review by Shen et al. (63). However, it is possible that the B cells within LAs function as well-placed antigen presenting cells to the surrounding T cells, as they express activation markers such as CD69, HLA-DR and CD83 to a higher degree than their peripherally derived counterparts (65). Over 70% of CD68^+^ endometrial macrophages are alternatively activated, identified by co-expression of CD163 (66). Alternatively activated macrophages, otherwise known as M2 macrophages, traditionally express anti-inflammatory cytokines such as interleukin (IL)-10 and participate in wound healing and tissue re-modeling (67). Major histocompatibility complex (MHC)-II, CD80 and CD86 expression on endometrial macrophages is low (66). As these proteins are involved in antigen presentation to CD4^+^ T cells, it is possible that endometrial macrophages have limited capacity to stimulate a CD4^+^ T cell response.

Collectively, our current understanding suggests that the healthy endometrium is biased towards a regulatory immune environment, while retaining the capacity to rapidly switch to traditional immune defense mechanisms due to the presence of classical type 1 effector cells. However, the majority of research on the immunological state of the normal, non-pregnant endometrium was conducted over 20 years ago, leaving room for confirmation of these findings with present-day technologies.

## Immunity in the cancerous endometrium

The relevance of the iTME for EC diagnosis and treatment is highlighted by the fact that EC molecular subtypes differ both in their tumor-infiltrating immune cell density and prognosis. CD8^+^ T cell infiltrates generally indicate a good prognosis (68–70), and advanced stage ECs have lower T cell density (71). The working hypothesis to support this finding is that tumors with fewer somatic mutations produce lower levels of immunogenic antigens. As such, these tumors can avoid detection by cytotoxic T cells and are more likely to advance to a late stage (72). Indeed, copy number low EC tumors have the second highest mortality rate (73). The correlations between molecular subtype and tumor-infiltrating immune cell abundance and phenotype have been investigated using IHC. A high proportion of CD8^+^ T cells in *POLE*mut and MMRd tumors was discovered, with most expressing the immunological checkpoint molecule, programmed cell death protein 1 (PD-1) (74). The binding of the PD-1 ligand, PD-L1, to PD-1 restricts T cell function, and is a key mechanism of immune tolerance in cancer (75). As such, high PD-1 expression by tumor-infiltrating T cells is a target for PD-1 inhibition by immune checkpoint inhibitors. Effective inhibition of PD-1 prevents the binding of PD-L1, ameliorating immune tolerance by the PD-1/PD-L1 pathway and elevating T cell efficacy (76). The PD-1/PD-L1 pathway is discussed in more detail in the section on targeting the immune microenvironment further on in this review.

The abundance of tumor-infiltrating immune cells in *POLE*mut and MMRd subtypes has been validated by multiple studies (74, 77, 78) and is likely to contribute to the comparatively high survival rates of these subtypes as well as their clinical responsiveness to immunotherapy. Indeed, MMRd tumors had significantly higher CD3^+^ cells expressing both PD-1 and PD-L1 compared to both NSMP and p53abn subtypes (79). While the advantage of PD-1 inhibition in high PD-1 expressing T cells is clear, the advantage of PD-L1 expression on T cells is more enigmatic. Recent evidence has elucidated a bi-directional role of PD-L1 expressed by tumor-associated T cells whereby it can act as both ligand and receptor. This gives T cells the ability to induce immunosuppressive phenotypes in other immune infiltrates such as macrophages, while also repressing its own differentiation into an effective anti-tumor cell (80). Furthermore, the tumor itself can induce T cell PD-L1 expression to enhance immune tolerance (80).

Tumor-infiltrating immune cells have been implicated as prognostic indicators in a range of cancers including melanoma, endometrial, ovarian, and colorectal cancer (81–83), and are positively associated with EC disease severity (77). The prognostic ability of an increase in particular immune subsets is less clear. For example, the number of regulatory T-cells (Treg) is both positively and negatively associated with overall survival in NSMP (84) and p53abn EC (77), respectively, suggesting a potential molecular subtype-specific role for these T cells. In parallel to significant T cell infiltration, EC is also characterized by an influx of CD3^-^CD56^+^ NK cells in tumor tissue compared to non-tumor tissue (85). Tumor-resident NK cells, as identified by the expression of CD103, have attenuated functionality as they express the inhibitory molecules TIGIT (T cell immunoreceptor with Ig and ITIM domains) and TIM-3 (T cell immunoglobulin and mucin-domain contaoining-3) (85). It is not known whether inhibitory signals from the tumor are causing this effect, or whether the cells are exhausted by other means, but it is likely that the result is immune tolerance of the cancer.

Tumor associated macrophages (TAMs) are a diverse subgroup of tumor-infiltrating immune cells derived from monocytes with traditionally cytotoxic and phagocytic attributes (86), but have also been implicated in cancer tolerance (87). It is therefore important to distinguish cancer tolerant TAMs from cancer intolerant TAMs to determine their prognostic significance. However, doing so is not simple. TAMs are traditionally defined as either M1 or M2, which represent the extremes of macrophages functional state – pro-inflammatory and regulatory, respectively. Importantly, these states are not mutually exclusive and as such this dichotomous nomenclature paradigm is beginning to shift to include additional (sub)categories of macrophage activation states (88). In EC, increased total TAM count, identified by positive IHC staining for CD68, was observed in both tumor and stromal tissues collected from EC patients compared to controls of benign pathology (89). However, there was no significant correlation between TAM density and cancer progression, so it remains unclear whether measuring TAM density in EC has any clinical or prognostic implications. Further investigation using IHC identified an increasing density of macrophages in the stromal compartment of EC as the severity of the disease increased, with fewer macrophages in the EC precursor endometrial hyperplasia, and the highest density observed in non-endometrioid EC, which tend to be more aggressive than EEC (90). However, the density of TAMs expressing CD163, a marker of M2 macrophages, was similar across EC histologies (90), suggesting that TAM infiltration in more aggressive EC tumors is predominantly comprised of the pro-inflammatory M1-like subtype. Conversely, co-culture of EC cell line-derived exosomes with a monocyte cell line can induce an M2-like macrophage phenotype (91), suggesting that tumor cells can polarize macrophages towards immune tolerance. Taken together, these data provide some evidence of the state and function of TAMs in EC but further investigation is warranted.

EC does not seem to be heavily infiltrated by B cells (74), but their role in EC disease progression warrants further investigation in light of recently described antigen-independent mechanisms (92). Specifically, the binding of dimeric IgA, but not monomeric IgG, to the polymeric immunoglobulin receptor (pIgR) initiates cell-intrinsic inflammatory, endoplasmic reticulum stress and pro-apoptotic pathways, thereby leading to improved patient survival. Albeit antigen-independent, this mechanism is of broad relevance to EC, and pIgR is quasi-universally expressed in EC cells. Additionally, tertiary lymphoid structures (TLS) are a major source of B cells in EC (93). TLS are similar to LAs in that they are comprised of a B cell core surrounded by T cells. They may play an important role in EC protection, as their absence is related to more progressive disease (93).

In summary, the iTME of EC is characterized by the infiltration of innate and adaptive immune cell subsets with anti-tumoral activity, and the molecular subtype of the cancer affects the immune infiltration. However, the literature is skewed towards describing the role of T cells in this context. Since the cytotoxic capacity of tumor-resident CD8^+^ cells is actively restricted by the iTME (94), the quantification of T cell subset or NK cell density is only meaningful if paired with established functional markers such as PD-1, TIM-3 or CD163. Furthermore, alongside the quantification of effector cells, it is important to consider the influence of regulatory immune cell subsets such as Tregs (95) and myeloid derived suppressor cells (96) on effector cell function. Pairing functional markers and regulatory immune subsets with immune cell density would give a more accurate picture of the interactions within the iTME, and thus would allow researchers to develop tools that work specifically to reduce the capacity of those that are advantageous to cancer progression.

## Hormone therapy in endometrial cancer

Endometrial cancer is commonly described as a hormone-driven cancer. This refers primarily to the most common subtype, EEC. As mentioned previously, prolonged exposure to high levels of bioavailable estrogen is the main driver of this histological subtype. The natural antagonist of estrogen is progesterone. This has prompted the use of levonorgestrel (LNG), a synthetic progestogen, as a novel treatment for early-stage EEC. While LNG administration is hormonal therapy, it is important to consider the immunological side-effects of such treatment, whether beneficial or detrimental. LNG activates the PR, binding with three times more affinity than natural progesterone (97). As discussed previously, the presence of PR in immune subsets is yet to be unequivocally determined. It is therefore important to note that LNG has an over 40-fold higher affinity to PR as compared to the glucocorticoid receptor (97) and would thus work more effectively on PR-expressing cells. The antagonistic effect of progesterone is also observed in immune cells, with progesterone-treated cells exhibiting an attenuated phenotype (23, 24). For example, progesterone causes the differentiation of CD4^+^ T cells towards a Th2 profile (23), which is generally regarded as anti-inflammatory. This mechanism is thought to be a major driver of pregnancy tolerance, as progesterone increases substantially during early pregnancy. To support this notion, progesterone can also dose-dependently reduce the activation status of human peripheral CD4^+^ T cells (24).

It is plausible that, alongside antagonizing estrogen, the use of LNG on early-stage EEC is dampening the immune response to EC. The downstream effects of this should be investigated as LNG treatment becomes more widely established. There is little information on the effect of LNG on endometrial immune cell populations. It appears that LNG has an immunoregulatory effect, with increased IL-10 expression by CD4^+^ and CD8^+^ T cells post-stimulation and significantly more endometrium-resident regulatory T cells in healthy LNG-IUS users compared to controls on no contraception (98). LNG also appears to reduce immunological surveillance, with fewer CD4^+^ and CD8^+^ endometrial T cells in LNG-IUS users compared to controls (98). Conversely, CD4^+^ and CD8^+^ T cells were more likely to express the activation markers CD38 and HLA-DR (98) indicating that, despite reduced numbers, the T cell compartment is in an increased state of activation with LNG-IUS use. While the argument has been made that the presence of a foreign body in the uterus results in a local inflammatory response (99), whether the observed increase of these activation markers is caused by a foreign body reaction or the LNG itself is not yet known. One study demonstrated that the endometrial transcriptome from LNG-IUS users exhibited more inflammatory markers and immune activation than those using the copper intra-uterine device, which was indistinguishable from non-IUS users (100). This would support the hypothesis that LNG regulates the endometrial immune compartment beyond a foreign body reaction. It must also be stated that, collectively, the studies discussed in this section up to this point investigate the effects of progesterone and LNG in the normal endometrial immune microenvironment. There is scope to explore whether these findings hold true of progesterone and LNG treatment within the iTME of EC.

## Obesity, immunology and endometrial cancer

The link between obesity and EC is well established. Obesity, defined by the World Health Organization (WHO) as a BMI > 30 (101), is the leading modifiable risk factor for EC. The rise in EC incidence has been directly related to the obesity epidemic (102). Between 40% to 60% of EC incidence in the United States and the United Kingdom has been ascribed to excess weight (103, 104). All measures of increased adiposity (waist-to-hip ratio, hip and waist circumference, weight gain and high BMI) increase the relative risk of EC development (105). Moreover, there is a positive association between increasing BMI and EC mortality, with a hazard’s ratio of 1.43 (confidence interval 1.26-1.61) per 5 kg/m^2^ increase in BMI (106). Bariatric surgery and the subsequent weight loss associated with it has been shown to reduce the relative risk of developing EC (107), which highlights the interconnectedness of increased body mass with this hormone-sensitive cancer. One of the molecular mechanisms explaining this relationship is the production of aromatase by adipocytes, which is an enzyme that cleaves androgens into estrogens (108). Increasing adiposity contributes more aromatase, and consequently more estrogen, to the endometrial environment, to directly promote endometrial cell proliferation. Obesity also reduces the amount of hormone-binding globulin, a carrier molecule which reduces the activity of estrogen molecules (108). These combined molecular processes result in elevated levels of bioavailable estrogen. Obesity also contributes to an increase in other EC risk factors including anovulation and polycystic ovarian syndrome, culminating in a higher risk profile for EC.

As obesity is associated with chronic inflammation (109), a recent study assessed the link between inflammation and weight loss on the endometrial iTME in participants classed as high-risk for EC due to having a BMI > 40 kg/m^2^. Participants received either bariatric surgery or a low-calorie diet to support weight loss. Blood and endometrial biopsies were taken to assess a range of immune markers using IHC and tissue imaging including CD68 (a pan-macrophage marker), CD56 (an NK cell marker), CD3 (a pan-T cell marker), CD8 (a cytotoxic CD8^+^ T cell marker), FOXP3 (a transcription factor of Tregs), and PD-1 (110). The authors found that weight and BMI were inversely correlated to CD8^+^ T cell infiltration but found no significant difference in any other immune subsets examined. This relationship between BMI and reduced CD8^+^ T cell infiltration in EC has been corroborated by another recent study (3). Thus, obesity specifically reduces endometrial immune surveillance by CD8^+^ cytotoxic T cells. This contrasts with what has been observed in peripheral blood of obese but otherwise healthy women, where CD8^+^ cell count was higher in obese compared to non-obese healthy women. Women with increasing BMI also had higher white blood cell counts in general than those in the normal index range (111). These findings suggest that increasing adiposity can reduce CD8^+^ T cell trafficking and tumor infiltration. Furthermore, CD8^+^ T cells from the tumor of obese MC38 mice produce less IFN-γ than non-obese mice (3). IFN-γ is a major anti-tumoral effector cytokine, thus demonstrating CD8^+^ T cell reduced functionality. Importantly, immune suppression and reduced tumor infiltration can be reversed with weight loss. In one case study, a patient with EC receiving an LNG-IUS underwent vertical sleeve gastrectomy for weight loss. Endometrial biopsies were analysed after 6 months. The patient lost 26 kg which correlated with an increase in CD3^+^ and CD8^+^ T cell tumor infiltration (3).

NK cells are known to be impaired in obese individuals, with fewer circulating and resident NK cells present with reduced anti-tumor functionality (112–114). Weight loss appears to ameliorate the effect (115). This evidence is in keeping with our current understanding of T cell behavior and distribution within the tumor. It is therefore likely that NK cells are similarly affected by increasing adiposity and could be driven by a change in tumor metabolism to increase consumption of fatty acids, which can have immunoregulatory effects (116). A conflicting study found no significant difference in peripheral NK cell number or cytotoxic capacity in lean compared to overweight or obese participants of similar age (117). This study opposes the current knowledge on NK cells and obesity and, as such, warrants further investigation.

Interestingly, increased BMI was found to positively correlate with the effectiveness of immunotherapy using pembrolizumab, an anti-PD-1 monoclonal antibody, in 36 PD-L1^+^ gynaecologic cancers including MSI high EC (118). PD-L1 positivity is an important control in this study as it means that BMI is the variable, not PD-L1 expression, making the data on increased BMI and efficacy of pembrolizumab more reliable. This data suggests that, although high BMI appears to suppress cytotoxic T cell capacity, immunotherapy may more effectively ameliorate the immunosuppressive iTME in individuals with higher BMI by overcoming adiposity-related immunosuppression. The mechanistic origin of the association between immunotherapeutic response and high BMI remains to be explored. However, it is intriguing to consider that obesity-associated intestinal hyperpermeability may prime immune effector functions by mediating the anticancer activity of ICIs through the circulation of bacterial metabolites, similarly to chemotherapy (119, 120). In summary, the immunosuppressive effect of obesity in EC could be a driver of immune-resistance in this highly obesity-associated cancer. Weight loss can reverse these effects and so should be incorporated into awareness campaigns and clinical management of EC. Increased BMI may be related to effective immunotherapy in this context, so further investigation is warranted here.

## Targeting the immune environment in endometrial cancer: Beyond checkpoint inhibition

Cancer immunotherapies involve targeting the immune system to drive a specific immunological function. This leads to greater outcomes in patients whose therapy can be tailored to their needs (121). Personalized medicine in the form of immunotherapies are gaining traction as adjuvant treatments for EC. These immunotherapies work best in *POLE*mut and MMRd tumor profiles due to their high mutation burden and associated increase in the production of immunogenic antigens (122). Despite this, the only immunotherapies currently FDA-approved are pembrolizumab and dostarlimab (both PD-1 inhibitors) for patients with MMRd and a pembrolizumab/lenvatinib (a multitargeted tyrosine kinase inhibitor) combination as a second-line therapy for MMR proficient patients (123). Immunotherapies for EC and the possibility for immune phenotypes to be used as prognostic indicators have been reviewed extensively elsewhere (79, 124–127). Here, we focus on the important role of the iTME for the therapeutic success of immunotherapies.

‘Immunotherapies’ technically include any therapy which targets the immune system to achieve a beneficial therapeutic outcome, but are mostly restricted to cancer vaccines, ICI therapies or passive infusion of cancer-specific T cells (124). Immune checkpoint inhibition, particularly the inhibition of the PD-1/PD-L1 (128) and cytotoxic T-lymphocyte-associated antigen 4 (CTLA4) pathways (129), together with TIM-3 pathway (130), have received great attention in the last decade and have put a spotlight on the immune system for treatment of cancer. These pathways, when engaged by ligands expressed by the tumor, provide inhibitory signals to the associated T cells with the exception of the engagement of TIM-3, which induces T cell apoptosis (129). The therapeutic success of ICIs depends on neoantigen immunogenicity and the presence of neoantigen-specific T cells, which are highly variable across patient populations. This limits the therapeutic efficacy against EC and is reflected by an overall response rate of only 13% in confirmed PD-L1 positive, albeit mostly non-MSI-H status, EC tumors with pembrolizumab treatment (131). Based on this, non-MSI-H tumors may be intrinsically less responsive to ICI than MSI-H tumors regardless of their PD-L1 expression pattern. Mechanistically, although PD-L1 expression might be high, which would indicate that the tumor is expressing immunosuppressive signals, T cell PD-1 expression may be low which would reduce the efficacy of the therapy. This is in line with the theory that immunotherapy works best in tumors with high mutational burden such as MSI-H, a term which can be used synonymously with MMRd. Indeed, a recent clinical trial in confirmed MMRd stage two or three rectal cancer patients found a 100% response rate after 6 months of treatment with another PD-1 inhibitor, dostarlimab (132). Although this study had a small sample size of just 12 patients and additional follow-up is needed to determine response in the remaining 4 patients who have not completed treatment, and to assess recurrence and response duration in those that do respond, this result is nevertheless a powerful indicator of how molecular subtype can be used to inform personalized treatment. Interestingly, recent *in silico* analyses unexpectedly demonstrated that MSI-H and non-MSI-H EC tumors are similarly infiltrated by immune cells (133), suggesting important functionality differences in the iTME between these EC subtypes. Highlighted here is the importance of assessing multiple parameters when implementing personalized treatment pathways, such as PD-1/PD-L1 positivity, MSI status and infiltrating immune cell phenotype and function.

MHC class I loss has been proposed as a possible mechanism of resistance to PD-1 inhibition (134) since this loss prevents neoantigen recognition by neoantigen-specific CD8^+^ T cells irrespective of PD-1 expression. MHC downregulation has indeed been documented in 42% of MMRd tumors (134). Therapeutic resistance to PD-1/PD-L1 ICI may also originate from the heterogenous expression of PD-1 in the iTME, and may be potentially overcome by targeting other inhibitory molecules such as TIM-3 as an addition or alternative to PD-1 targeting (135). Therefore, further categorisation of the specific deficiency of the MMRd tumor may be necessary to facilitate optimal treatment. TIM-3 expression is differentially expressed in immune infiltrates of different EC subtypes, with preferential expression in MMRd as compared to MMR intact tumors (136). However, more work is required to delineate the respective contributions of tumor versus immune cell specific TIM-3 expression and their relevance for TIM-3 targeted ICI. As TIM-3 expression on cancer cells predicts response to PD-1 targeted ICI in other solid cancers (137), the consistent expression of TIM-3 on both MMRd and MMR intact tumor cells (136) suggests that ICI approaches combining PD-1 and TIM-3 (138) may provide therapeutic benefit in EC irrespective of subtype or mutational burden. Additionally, the inhibition of CD47, a ligand for the macrophage-associated signal-regulatory protein α (SIRPα) has been shown to increase phagocytosis of EC tumor cells by TAMs (139), although CD47- SIRPα pathway inhibition has not yet been considered for clinical trial.

Importantly, an integrative approach to immunotherapy against EC requires the consideration of hormonal therapy and its strong influence on the iTME. As a number of conventional cancer treatment modalities such as radiotherapy and chemotherapy have been described to indirectly harness the immune system (120, 140), it is conceivable that LNG treatment may also play a significant role in future approaches to EC immunotherapy. Comparably, androgen deprivation therapy has been used as an immunotherapy in prostate cancer, whereby blocking the androgen receptor ameliorates T cell function by increasing CD8^+^ T cell expression of the pro-inflammatory cytokine, IFN-γ (141). We have described LNG’s intrinsic immunosuppressive activity in a previous section, suggesting that treatment with LNG may reduce the capacity of the iTME to control cancer growth. However, such effect could be partly counteracted by LNG-mediated early EC cell death by starving EC of bioavailable estrogen, which enhances cancer-associated antigen presentation and immune responses, a mechanism previously described for radiotherapy (142).

## Discussion

Due to the inconsistent classification system of EC, the work examined in this review largely uses either histological or molecular subtype, but not both. As it is impossible to infer a histological subtype based on a molecular subtype and vice versa, interpreting the role of infiltrating immune cell proportion and phenotype within EC subtypes across studies is difficult. Future research, both within the scope of understanding the iTME of EC and beyond, would benefit from incorporating both histological and molecular classifications. Although this is a more costly exercise, it may facilitate discoveries and is likely to be pivotal for personalizing treatment. It would be futile to investigate the iTME in EC and not comment on EC subtype, particularly molecular subtype, which can directly influence the iTME. Additionally, many studies are retrospective in design, utilizing publicly available TCGA data. Prospective studies would allow for more versatile experimental conditions, such as how therapeutics influence the immunologic phenotype and affect EC pathogenesis. Current literature is overwhelmingly skewed towards the immune profiles of MMRd and *POLE*mut tumors, however we understand very little about the iTME of the other molecular subtypes which may not be classed as immunogenic but could benefit from immunotherapy. Additionally, there is conflicting evidence regarding the clinical relevance of immune infiltration in patients with high versus low BMI, as well as the mechanism of the observed superior response to immunotherapy in high BMI patients. Trialing methods of immunotherapy other than the T cell PD-1/PD-L1 pathway, such as TIM-3 or the macrophage-associated CD47-SIRPα pathway, could expand the clinical arsenal of immunotherapy. Macrophages constitute up to 20% of CD45^+^ cells in the benign endometrium and, although there is little information on their presence in EC, it is feasible that their phagocytic capabilities could be harnessed for antitumor response.

Additionally, there is a large body of work from the 20^th^ century regarding the immunogenic state of non-malignant endometrium that needs to be verified using present day technologies. We are yet to unequivocally determine the expression of PR on immune cells and are therefore unable to investigate the specific molecular pathways induced by progesterone, and its derivatives, in these cells. LNG is currently being used as a contraceptive and therapeutic for early-stage EEC, yet its effect on the endometrial immune landscape remains largely unexplored. The little evidence we have suggests that LNG, although effective in some cases at inhibiting EC growth by restricting responsiveness of tumor to estrogen, may contribute to an immunosuppressive state. It is crucial that the effect of LNG on endometrial-resident immune cell subsets be explored further to ensure that any detrimental effects on the immune landscape are included in the risk assessment of the treatment when used as either a contraceptive or cancer therapeutic.

Immune cell count and proportion have both been investigated as prognostic markers for EC, but evidence is needed to identify which proportions of immune cells confer prognostic advantages or disadvantages in each EC subtype before these measurements can be of clinical benefit. A comprehensive review of studies examining the tumor infiltrating immune cells of EC and clinical outcome would consolidate the research and provide a framework for new research opportunities. Furthermore, literature investigating infiltrating immune cells in EC is predominantly restricted to the role of T cells. Although some have identified a role for NK cells, tumor associated macrophages (TAMs) and B cells, further research into the participation of non-T-cell immune cells is warranted. NK cells are well known for their cytotoxic capacity and are a major part of the iTME. Their importance, as well as the role of other immune subsets, should not be overlooked. While evidence suggests a major role of CD8^+^ cytotoxic T cells in adiposity-driven immune suppression in EC, there is controversy regarding the role of NK cells in this context. Additionally, although we understand the role of obesity in driving immune suppression in non-EC participants, the role of immunity in obesity-related EC pathogenesis is yet to be explored.

The immune landscape of endometrial cancer remains incompletely understood. In the non-cancerous endometrium, immune cells are under the influence of cyclic hormonal regulation. At this time, the dominating immune phenotype appears to be regulatory. When the endometrium becomes malignant, many immunological changes are induced which tend to vary based on the molecular classification of the cancer. As such, molecular classification of EC should be incorporated into standard-of-care practices, as well as future research. Furthermore, EC is increasingly associated with obesity, the combination of which is typified by fewer tumor infiltrating cytotoxic T cells with limited cytotoxic capacity. Importantly, this can be reversed with weight loss. Novel treatments for EC such as LNG-IUS and ICIs have (potentially opposing) direct impacts on the iTME and gaining a deeper understanding of the immunological mechanisms of these treatments could lead to personalized, novel treatments and treatment regimens. Progesterone has immunosuppressive effects. Hormonal treatment of inoperable early-stage EEC using LNG-IUS can be effective in preventing EC proliferation, but its immunosuppressive effects could contribute to the low response rates observed for this therapy. Immunotherapies for adjuvant treatment of EC are still in their infancy and show promise for molecular subtypes characterized by high immune infiltration, but their use does not necessarily need to be restricted to such subtypes. A focus on characterizing the response to immunotherapies across the spectrum of subtypes is likely to yield important insight into their effectiveness in previously understudied subtypes. We recommend exploring novel mechanisms of immune activation and exhaustion, for example investigating immunotherapies targeting macrophages so as not to limit our capacity for immune manipulation to the PD-1/PD-L1 pathway.

## Author contributions

HV wrote the first draft of the manuscript. KH, MC, OG, and CH edited sections of the manuscript. All authors contributed to manuscript revision, read, and approved the submitted version.

## Acknowledgments

**Figure 1** was created with Canva.com. **Figure 2** was created with BioRender.com.

## Conflict of interest

The authors declare that the research was conducted in the absence of any commercial or financial relationships that could be construed as a potential conflict of interest.

## Publisher’s note

All claims expressed in this article are solely those of the authors and do not necessarily represent those of their affiliated organizations, or those of the publisher, the editors and the reviewers. Any product that may be evaluated in this article, or claim that may be made by its manufacturer, is not guaranteed or endorsed by the publisher.
